# Does Whole-Body Hypothermia in Neonates with Hypoxic–Ischemic Encephalopathy Affect Surfactant Disaturated-Phosphatidylcholine Kinetics?

**DOI:** 10.1371/journal.pone.0153328

**Published:** 2016-04-12

**Authors:** Matteo Nespeca, Chiara Giorgetti, Stefano Nobile, Ilaria Ferrini, Manuela Simonato, Giovanna Verlato, Paola Cogo, Virgilio Paolo Carnielli

**Affiliations:** 1 Division of Neonatology, Department of Clinical Sciences Polytechnic University of Marche and Azienda Ospedaliero Universitaria Ospedali Riuniti, Ancona, Italy; 2 Department of Women’s and Children’s Health, University of Padova, Padova, Italy; 3 Pediatric Research Institute “Città della speranza”, Padova, Italy; 4 Department of Experimental and Clinical Medicine, University of Udine, Udine, Italy; Centre Hospitalier Universitaire Vaudois, FRANCE

## Abstract

**Background:**

It is unknown whether Whole-Body Hypothermia (WBH) affects pulmonary function. In vitro studies, at relatively low temperatures, suggest that hypothermia may induce significant changes to the surfactant composition. The effect of WBH on surfactant kinetics in newborn infants is unknown. We studied in vivo kinetics of disaturated-phosphatidylcholine (DSPC) in asphyxiated newborns during WBH and in normothermic controls (NTC) with no or mild asphyxia. Both groups presented no clinically apparent lung disease.

**Methods:**

Twenty-seven term or near term newborns requiring mechanical ventilation were studied (GA 38.6±2.2 wks). Fifteen during WBH and twelve NTC. All infants received an intra-tracheal dose of ^13^C labelled DSPC and tracheal aspirate were performed. DSPC amount, DSPC half-life (HL) and pool size (PS) were calculated.

**Results:**

DSPC amount in tracheal aspirates was 0.42 [0.22–0.54] and 0.36 [0.10–0.58] mg/ml in WBH and NTC respectively (p = 0.578). DSPC HL was 24.9 [15.7–52.5] and 25.3 [15.8–59.3] h (p = 0.733) and DSPC PS was 53.2 [29.4–91.6] and 40.2 [29.8–64.6] mg/kg (p = 0.598) in WBH and NTC respectively.

**Conclusions:**

WBH does not alter DSPC HL and PS in newborn infants with no clinical apparent lung disease.

## Introduction

Whole-Body Hypothermia (WBH) treatment has become the standard of care for neonates with moderate or severe hypoxic-ischemic encephalopathy (HIE) [1–4]. Most of the interest focused on brain function and reletively little information has been gained on the effect of WBH on other body organs and more so on the lungs [5].

Respiratory failure is often encountered in asphyxiated newborns who receive WBH, ranging from 26% to 39%, and it can be due to different causes such as Meconium Aspiration Syndrome, Persistent Pulmonary Hypertension of the Neonate, Pulmonary Hemorrhage, congenital pneumonia and Idiopathic Respiratory Distress Syndrome [6,7]. In most cases the etiology of the lung dysfunction remains undefined, since the specific mechanism of lung damage is not clearly defined and multiple factors probably are mutually reinforcing the lung injury in each infant [6]. To the best of our knowledge there are no information on pulmonary surfactant function in humans, when the body temperature is maintained at 33.5°C for 72 hours. Lowering body temperature could alter surfactant fuction from one side, or conversely it could blunt the inflammatory process associated with neonatal asphixia.

Dassios *et al* reviewed the data of 31 mechanically ventilated full-term newborn who underwent WBH for HIE. They observed an improvement in oxygenation and ventilator parameters during induction of hypothermia [8]. Recent data showed better respiratory outcomes and trends towards lower inflammation in WBH-treated preterm lambs, suggesting a possible role of hypothermia in reducing lung injury through the modulation of the inflammatory pathway [9]. De Luca *et al*. have recently reported significant reductions in IL-6 and IL-8 levels in Epithelial Lining Fluid during WBH [10].

Experiments in hibernating animals showed temperature induced adaptive changes to surfactant composition and structure at temperature ranging from 37°C to 3°C [11]. The surface reducing tension of pulmonary surfactant depends, not only, on surfactant amount and composition but also on its lipid bilayer structure and dynamics that are affected by temperature [12]. There is *in vitro* evidence that hypothermia induces changes in surfactant phospholipid composition. The presence of a higher proportion of unsaturated phospholipids reduces the melting temperature of surfactant membranes leading to the transition from a liquid-crystalline phase to the gel-phase [13]. Howard *et al*. in an *in vitro* study, reported that lowering the temperature from 20°C to 5°C of Dipalmitoyl-Phosphatidylcholine (DPPC) vesicles in water results into an increased aggregation which can be reversed by an increase in temperature [14]. Recent data in neonates with HIE requiring WBH reported that the interfacial absorption of surfactant showed no changes after 24h of hypothermia (33.5°C) whereas a significant reduction occurred after 72h.

Nevertheless, no data are currently available on surfactant metabolism *in-vivo* in asphyxiated newborn infants during WBH.

DSPC accounts for at least 40% of pulmonary surfactant phospholipids and it plays a critical role on surfactant function since this lipid alone accounts for the surface tension-lowering properties that prevent alveolar collapse and is essential for normal lung gas exchange [15]. As hypothermia might affect lipid organisation and phospholipid surfactant composition we designed, as first step a preliminary study to investigate Disaturated-Phosphatidylcholine (DSPC) kinetics in vivo in asphyxiated newborns without pulmonary disease.

## Materials and Methods

### Subjects

We conducted a prospective pilot study in the Neonatal Intensive Care Unit of the Polytechnic University of Marche-Ancona and of the University of Padova, Italy from 2009 to 2013. Among the 67 newborns who underwent WBH during this period in the two centers, we selected 15 newborns with no lung disease to perform the surfactant DSPC kinetic study. Fifty-two subjects were excluded for different reasons than lung disease (short duration of study period, anavailable tracer, increase of FiO2 during hypothermia, informed consent not obtained, etc). Eligible infants were late-preterms (35 to 36 completed weeks of gestation) or term newborns (37 to 41 completed weeks of gestation) having moderate or severe perinatal asphyxia and undergoing WBH according to TOBY criteria (3). We enrolled newborns who fulfilled the following inclusion criteria: 1) normal chest radiograph; 2) prediction to be mechanically ventilated for at least 48h; 3) FiO_2_ less than 0.24 to achieve an arterial oxygen saturation > 95% for more than 96% of the study period. Normothermic controls (NCT) patients consisted of 12 term newborns who required mechanical ventilation for major surgery, myopathy or neurologic failure. In the NTC group, infants were kept normothermic (37.0±0.5°C) during the surfactant DSPC kinetic study.

In our neonatal unit therapeutic hypothermia is considered standard of care for infants with moderate to severe hypoxic ischemic encephalopathy (HIE), thus it is nearly impossible to have as control group infants with asphyxia (moderate to severe) requiring mechanical ventilation and not requiring hypothermia. Exclusion criteria for both groups were evident pulmonary diseases, such as Meconium Aspiration Syndrome, pulmonary hemorrhage, signs of infection, congenital malformations, metabolic disorders, chromosome abnormality, congenital infections, liver and renal failure, exogenous surfactant administration and lack of parental consent.

### Study design

As soon as the informed consent was obtained from both parents, study patients received an intratracheal tracer dose 1ml/Kg (2 mg/kg) of (U-^13^C-PA)—DPPC (Avanti Polar Lipids, Alabaster, AL) mixed with 8 mg/kg (0.1 ml/Kg) of porcine surfactant extract (Curosurf®, Chiesi Pharmaceuticals, Parma, Italy) as spreading agent. We administered the tracer via a small catheter inserted through the endotracheal tube at the level of the carina; in the WBH group, the tracer was administered at the hypothermic target temperature. Stable isotope labelled DPPC was prepared as previously described [16–19] in a sterile way by the hospital pharmacy and the delivering procedure was performed with no side effects. We collected tracheal aspirates at baseline before tracer administration (t = 0), every 6 hours for the first 72 hours and then every 12 hours for other 2 days or until extubation.

Baseline aspirate was collected usually during passive hypothermia or after fewer hours from the beginning of active hypothermia due to the time necessary to obtain parent’s informed consent.

Tracheal aspirates were performed during routine patient care and the frequency of suctioning did not deviate from the normal clinical practice at both study centers. Criteria to start WBH for both centers followed the TOBY study criteria [20]. Neonates were continuously kept at an esophageal temperature of 33.5 ± 0.5°C for 72 hours using a whole body servo-controlled mattress (Criticool®, Vital care products, Inc, Milan, Italy).

According to local guidelines, neonates were mechanically ventilated until rewarming. Rewarming was performed at a rate of 0.5°C per hour until the body temperature reached 36.5°C. We recorded clinical data, vital and ventilator parameters hourly or more often when indicated. The monitoring schedule, including arterial gas analysis, was prescribed by the attending physician depending on patient’s clinical needs and independently from the study, thus the study did not require any change of the routine clinical practice, apart from the administration of the tracer. The protocol was approved by the Ethics Committee of the University of Padova and by the Ethics Committee of the Polytechnic of Marche, and written informed consent was obtained from both parents of each infant. No external funding sources were granted for this study. Parents were informed that their child was not going to benefit from the study, but that the study results could potentially help other infants with the same clinical condition in the future.

### Tracheal aspirate collection and storage

Tracheal aspirates were performed as previously described [18,21]. Briefly, after instillation of 1 ml/kg saline (0.9% NaCl) in the endotracheal tube, the neonate was gently hand-bagged and then tracheal secretions were collected through a Lukens trap at the pre-set time points. Samples were stored at 4°C for no longer than 1 h and brought to a final volume of 2 ml with 0.9% saline. Tracheal aspirates with visible blood were discarded. After gentle vortexing, samples were centrifuged at 400 g for 10 minutes. Supernatants were aliquoted and stored at -80°C, until analysis.

### Measurement

Lipids from tracheal aspirates were extracted according to Bligh and Dyer [22], lipid extracts were oxidized with osmium tetroxide and DSPC was isolated from the lipid extract by thin layer chromatography as previously reported [23]. The DSPC fatty acids were derivatized as methyl-ester and extracted with hexane [19]. DSPC fatty acid composition was analyzed by gas-chromatography-FID and the ^13^C enrichment of DSPC-palmitate by gas-chromatography-mass spectrometry (GC-MS, 6890N-5973 inert, Agilent Technologies, Milan, Italy). Enrichment was expressed as mole percent excess (MPE) and calculated using a calibration curve.

### Data analysis

Surfactant DSPC half life (HL) and DSPC pool size (PS) were calculated from the exponential decay of the ^13^C palmitate-DSPC enrichment curve; this method has been previously described in detail [16]. Briefly, the biological HL of surfactant DSPC is the time required for one half of the tracer (labelled DSPC) to be removed from the alveoli whereas DSPC pool size is calculated form the dilution of the tracer in the tracee pool (endogenous DSPC). The amount of DSPC from tracheal aspirates was given as the mean value of tracheal aspirates of each individual study patient. Data were expressed as mean ± SD or as median [IQR] according to variable distribution. Comparisons were made by Student t-test and Mann-Whitney Test, as appropriate for continuous variables, and by Chi-square for categorical variables. A p<0.05 was regarded as statistically significant. Statistical analysis was performed using PASW Statistics 19.0 for Windows (SPSS Inc., Chicago, IL). No preliminary data on DSPC HL and PS were available to calculate sample size.

## Results

We studied 27 term and near term infants without apparent lung disease: 15 asphyxiated infants were treated with WBH and 12 were nursed in a thermo-neutral environment. Clinical characteristics of study infants are reported in Table 1. In the NTC group, 7 infants had abdominal surgery requiring prolonged sedation and mechanical ventilation, 3 had congenital myopathies and 2 had perinatal asphyxia but with no indication for therapeutic hypothermia. As expected, the mean body temperature was significantly different between the two study groups. NTC infants were studied at an older age than the infants in the WBH group. The age difference between the two study groups is due to the fact that hypothermia, in asphyxiated patients, must be started during the latent phase of neural injury, ideally within the first 6 hour after ischemia, whereas 7 out of 12 control infants required mechanical ventilation for non-pulmonary diseases, like elective abdominal surgery, that occurs after the first days of life. The age range is wide also in infants treated with WBH but in all WBH subjects duration of kinetic study was at least 48 hours during active hypothermia, this allowed us to calculate accurate kinetic parameters.

**Table 1:** Clinical characteristics of study patients. NS: not significant, *P* < 0.05 was considered as statistically significant.

	WBH	NTC	*P*
Patients^a^ (N)	15	12	
GA^b^ (wks)	39.1 ± 1.3	37.9 ± 2.9	NS
Birth weight^b^ (g)	3113 ± 323	3089 ± 713	NS
Gender^a^ (male/female)	7/8	9/3	NS
Age at study start^c^ (d)	0.93 (0.66–1.29)	3.32 (2.16–17.2)	0,001*
Apgar Score at 5 min^c^	6 (5–10)	8 (5–9)	NS
Survival to discharge^a^ (N alive/N death)	14/1	10/2	NS
Centre^a^ (N centre 1/N centre 2)	13/2	7/5	NS
Temperature^b^^,^^d^ (°C)	33.28 ± 0.15	36.90 ± 0.14	0.000

^a^ Data presented as numbers. Values are compared with Fisher’s Exact Test.

^b^ Data are expressed as mean ± SD. Values are compared with independent samples t-test.

^c^ Data presented as median (IQR). Values are compared with Mann-Whitney test.

^d^ Temperature is expressed as degree Celsius and it is an esophageal temperature in the WBH and a cutaneous temperature in NTC.

**Fig 1:**
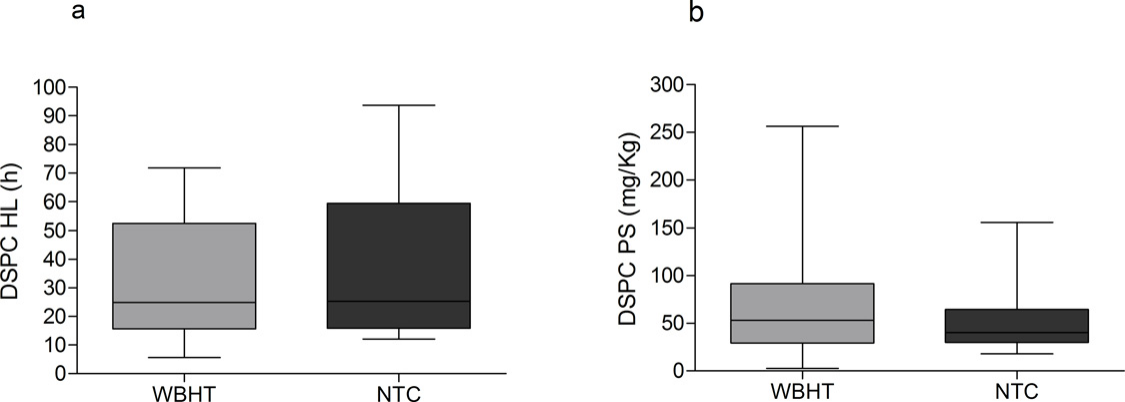
DSPC half-life and pool size in asphyxiated newborns under Whole-Body Hypothermia and in normothermic controls. Panel a: Median DSPC HL in the two groups, expressed in hours (h). DSPC HL was not significantly different in the WBH group compared with NTC group. Panel b: Median DSPC PS in the two groups, expressed in mg/kg. DSPC PS was not significantly different in WBH group compared to NTC group. Data were expressed as median (IQR).

There were no other significant differences between the two groups in terms of mean Fraction of Inspired Oxigen (FiO_2_), Mean Airway Pressure (MAP), Oxygenation Index (OI) and, PaO_2_/FiO_2_ ratio during the study period (Table 2). Mean FiO_2_ during study period was 0.22 ± 0.01 and 0.22 ± 0.02 in WBH and NTC respectively (p = 0.352). All study infants were on Synchronized Intermittent Mandatory Ventilation (SIMV) or SIMV/CPAP (Continuous Positive Airway Pressure) via an endotracheal tube. None of the patients received surfactant replacement therapy. The median amount of DSPC in tracheal aspirates during the study period was not significantly different between the two study groups, being 0.42 [0.22–0.54] mg/ml in the WBH group and 0.36 [0.10–0.58] mg/ml in the NTC group, p = 0.578. Surfactant DSPC kinetics could be reliably calculated in all study patients. Median DSPC HL was not significantly different between the two study groups, being 24.9 [15.7–52.5] h in the WBH group and 25.3 [15.8–59.3] h in the NTC group, p = 0.733 (Fig 1 panel A). Similarly, median DSPC PS was not significantly different in WBH infants compared to NTC newborns, being 53.2 [29.4–91.6] mg/kg of body weight and 40.2 [29.8–64.6] mg/kg, respectively, p = 0.598 (Fig 1 panel B).

**Table 2:** Ventilatory parameters of study patients. FiO_2_, fraction of inspired oxygen; MAP, mean airway pressure; OI, oxygenation index; PaO2, arterial pO2; NS, not significant, *P* < 0.05 is considered as statistically significant.

	WBH	NTC	*P*
FiO_2_^a^	0.22 ± 0.01	0.22 ± 0.02	NS
MAP^a^ (cmH_2_O)	5.2 ± 1.6	4.7 ± 1.7	NS
OI^a^	2.1 ± 1.4	1.9 ± 0.9	NS
PaO_2_/FiO_2_^a^	324 ± 130	247 ± 45	NS

^a^ Data presented as mean ± SD, referred to the study period. Values are compared with Independent samples T-test.

## Discussion

In this study we measured for the first time surfactant DSPC kinetics in term and near term infants without pulmonary disease during WBH and we found no difference between WBH and normothermic control infants. We used a state of art method, based on safe stable isotopes tracers [24], as previously reported by our group [16,17,21] that assesses the DSPC kinetics, the most abundant surfactant component, by using an intra-tracheal stable isotope labeled tracer. Strengths and limitations of this method have been previously discussed in other publications by our group [17].

Our findings strongly suggest that the DSPC turnover in term or near term newborns with no lung disease undergoing WBH is comparable to normothermic controls. This information is novel and we show that hypothermia is not responsible of alterations of DSPC surfactant turnover, at least in infants who do not develop respiratory failure in associatin with neonatal asphyxia, according to the study inclusion criteria. We chose to study patients with normal lungs as a first step in this line of research, because we felt it was important to obtain some “baseline information” on the effect of hypothermia on DSPC kinetics. DSPC PS, estimated *in vivo* by measuring the dilution of the DSPC tracer, was not significantly different between groups. However the different postnatal age could be responsible of a median value of DSPC PS that is 25% higher in the WBHT group than in NTC group. This difference is not significant and probably is due to the small number of study infants and to the wide variability of the pool size measuremets. This hypothesis is supported also by the fact that the amounts of DSPC from tracheal aspirates was not different between groups. Alternatively, it could be hypothyzed that the adrenergic stimuli may induce surfactant mobilization and production from type 2 pneumocytes. De Luca *et al*., studied pulmonary surfactant activity *by* fluorescence analysis of adsorption and found a significant increase in surfactant function after 48 h of WBH in neonates with HIE and no injured lungs, suggesting that adaptive mechanisms could act in surfactant modulation [10]. More information and studies designed to measure surfactant composition and production during systemic hypothermia may help understanding the effect of this treatment in severe lung injury [25,26].

Limitations of the present study are: first the small number of normothermic controls with asphyxia, if we had more, we could discriminate the effect of WBH from that of the hypoxic-ischemic insult; second, our control infants were on mechanical ventilation for major surgery, neuromuscular causes or neurological impairment. Although we acknowledge that the “best controls” would have been spontaneously breathing infants at room air, our study provides the best possible estimation of DSPC kinetics in humans with healthy lungs. These preliminary results are important for the next step of our project in which we will investigate the effect of WBH on surfactant composition and turnover in neonates with severe lung injury.

## Conclusions

In summary the present study examined the kinetics of DSPC in late preterm and term newborns with healthy lung during WBH. DSPC HL and PS did not significantly differ by lowering body temperature to 33.5°C compared to normothermic controls.

Further studies are needed to evaluate the kinetics of DSPC during WBH in case of lung injury.
